# The Financial Impact of Genetic Diseases in a Pediatric Accountable Care Organization

**DOI:** 10.3389/fpubh.2020.00058

**Published:** 2020-02-28

**Authors:** Katherine E. Miller, Richard Hoyt, Steve Rust, Rachel Doerschuk, Yungui Huang, Simon M. Lin

**Affiliations:** ^1^Research Information Solutions and Innovation, The Research Institute at Nationwide Children's Hospital, Columbus, OH, United States; ^2^Partners for Kids, Nationwide Children's Hospital, Columbus, OH, United States; ^3^Department of Biomedical Informatics and Department of Pediatrics, College of Medicine, The Ohio State University, Columbus, OH, United States

**Keywords:** genetic disease, computational phenotyping, pediatrics, accountable care organization, insurance claims

## Abstract

**Background:** Previous studies revealed patients with genetic disease have more frequent and longer hospitalizations and therefore higher healthcare costs. To understand the financial impact of genetic disease on a pediatric accountable care organization (ACO), we analyzed medical claims from 2014 provided by Partners for Kids, an ACO in partnership with Nationwide Children's Hospital (NCH; Columbus, OH, USA).

**Methods:** Study population included insurance claims from 258,399 children. We assigned patients to four different categories (1-A, 1-B, 2, & 3) based on the strength of genetic basis of disease.

**Results:** We identified 22.7% of patients as category 1A or 1B- having a disease with a “strong genetic basis” (e.g., single gene diseases, chromosomal abnormalities). Total ACO paid claims in 2014 were $379M, of which $161M (42.5%) was attributed to category 1 patients. Furthermore, we identified 23.3% of patients as category 2- having a disease with a suspected genetic component or predisposition (e.g., asthma, type 1 diabetes)- whom accounted for an additional 28.6% of 2014 costs. Category 1 patients were more likely to experience at least one hospitalization compared to category 3 patients- those without genetic disease [odds ratio [OR] = 4.12; 95% confidence interval [CI] = 3.86–4.39; *p* < 0.0001]. Overall, category 1 patients experienced nearly five times the number of inpatient (IP) admissions and twice the number of outpatient (OP) visits compared to category 3 patients (*p* < 0.0001).

**Conclusion:** Nearly half (42.5%) of healthcare paid claims cost in 2014 for this study population were accounted for by patients with single-gene diseases or chromosomal abnormalities. These findings precede and support a need for an ACO to plan for effective healthcare strategies and capitation models for children with genetic disease.

## Introduction

Accountable Care Organizations (ACOs) are groups of healthcare providers who collectively accept financial and clinical risk for defined patient populations. It is important to study and understand ACO payment models and how specific diseases or groups of patients affect payment distribution. Lowering medical spending is a primary goal of ACOs (1, 2). To bend the cost curve of ACOs, previous studies investigated care coordination, utilization management, and pharmacy management (3–5).

In adult ACOs, significant amount of spending is associated with chronic conditions, including heart disease, diabetes, and arthritis, conditions frequently associated with aging (6–8). For obvious reasons, pediatric ACOs face a fewer number of patients with these types of chronic conditions. For a pediatric ACO to improve value and quality of care, where should they focus their efforts?

Notably, although a specific genetic disease can be relatively rare, collectively, children with genetic diseases account for a disproportionately large percentage (nearly 10–20%) of pediatric hospitalizations (9–12). Studies using electronic health records and hospital discharge data report these children account for an even greater proportion of healthcare costs (10, 13, 14). One study reported nearly 70% of patients admitted to Rainbow Babies and Children's Hospital (Cleveland, OH, USA) had a known or suspected genetic disorder and accounted for >80% of annual healthcare costs in 1996 (10).

Genetic disorders collectively encompass Mendelian (single gene) diseases, chromosomal abnormalities, birth defects, or other congenital anomalies. To date, there are 6000+ known genetic disorders cataloged in Online Mendelian Inheritance in Man (15). The number of diseases with a known genetic component is rapidly increasing through the advancement of genetic research and breakthrough of sequencing technologies (16–18). Many birth defects or congenital anomalies have a known or suspected genetic basis; furthermore, there is increasing knowledge on the genetics of complex diseases, such as pediatric cancer and type I diabetes (19–23).

Collectively, the impact of genetic disease on healthcare costs and utilization specifically within a pediatric ACO has not been studied. We conducted a retrospective analysis of medical claims data to characterize the financial burden of genetically determined diseases, specifically in a pediatric ACO population in Central and Southeastern Ohio, USA.

## Materials and Methods

### Study Population and Sources of Data

**Figure 1:**
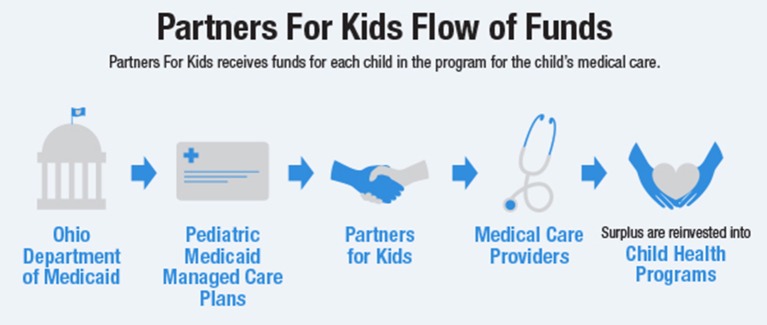
Partners for Kids Flow of Funds: PFK receives funds to pay for each child's medical care, the amount of which is defined as a capitation rate per child. A cost-saving model can achieve a surplus of funds, which are reinvested into programs that lead to improved health for children, such as school-based clinics and neighborhood programs.

We received study approval for this study from Nationwide Children's Hospital (NCH) Institutional Review Board (IRB), with a waiver of informed consent. We requested medical insurance claims data via a data use agreement, from Partners for Kids (PFK), a pediatric ACO in partnership with NCH (24). PFK is one of the nation's oldest and largest ACOs and acts as a bridge between the state of Ohio's five Medicaid Managed Care Plans and the care received by ~330,000 children in central and southeastern Ohio (Figure 1). We analyzed claims data for children (age 0–18) enrolled in PFK with continuous eligibility for 2014, excluding children with non-continuous eligibility or a lapse in coverage during 2014. In total, 258,399 patients were included in our analysis.

Six relational files came from PFK. Three claims files (inpatient, outpatient, and professional) were all linked by a unique encounter ID, which is specific for a given patient on a given date with a given provider. Each line of data for each patient included corresponding costs for an encounter. The remaining three files included a diagnosis file (International classification of diseases-9 (ICD-9) codes), a pharmacy file (prescription costs), and an eligibility file (demographics). All files included a unique member ID per patient, allowing us to aggregate all files to produce details on each patient for annual costs, number and types of visits, and length of stay for any hospitalizations.

### Study Design and Genetic Categorization

Similar to McCandless et al., we classified PFK members into four categories (1-A, 1-B, 2, 3) based on the presence of an underlying disease, and the extent to which that disease was genetically determined. A complete list of the diagnoses and *International Classification of Diseases, Ninth Revision* (ICD-9) codes used for our categorization is available in Supplementary Table 1. Category 1 includes children with disorders with a strong genetic basis; we further refined into subcategories to distinguish between clearly single gene/chromosomal disorders (1-A) and birth defects/congenital anomalies for which current literature and clinical experience suggest a strong genetic cause (1-B). Category 2 includes acquired disorders with a suspected genetic component or predisposition, while category 3 includes all children who were not classified as 1-A, 1-B, or 2. Patients were assigned to one category only and received the lowest numerical assignment (i.e., highest genetic designation) determined by their history of medical diagnoses. For example, patients were considered category 1-A if they ever had an ICD-9 diagnosis code within 1-A, regardless of any category 1-B or 2 diagnoses. PFK claims data containing ICD-9 diagnoses was available only from 2008 to present; we did not have diagnosis history for our patients prior to 2008.

For each category, we calculate the mean annual paid amounts per child, number of inpatient (IP) admissions (including emergency room (ER) admissions), total paid amounts for IP admissions, mean length of stay (LOS) per IP admission, number of outpatient (OP) visits (including office visits and non-admit ER visits), total paid amounts for OP visits, and total prescription (Rx) paid amounts. All monetary values used for analysis are the dollar amount of “paid claims,” as paid by PFK.

When we analyzed number of visits or admissions per child, we excluded patients with zero visits or admissions. For example, we did this to allow for accurate identification of three independent drivers for higher IP costs:

1. A higher percentage of category 1-A patients had ≥1 admission
2. Among those with ≥1 admission, category 1-A patients had more admissions (than categories 1-B, 2, or 3)
3. Among those with ≥1 admission, category 1-A patients had higher costs per admission

Had we included the patients with zero admission in the “admissions per child” analysis, drivers 1 and 2 become confounded. This same logic applies to our analysis of OP-office visits per child and OP-ER visits per child in the following sections.

### Statistical Analysis

Analyses were performed using R version 3.1.1 “stats” package and JMP 13.0.0 (SAS Institute, Cary, NC, USA) software. Descriptive statistics (mean and Bonferroni confidence intervals) of annual costs (paid claim amounts), number of visits, and LOS for each genetic category were calculated. Initial analyses were performed using 1-way analysis of variance; individual pairwise means comparisons between categories were performed using the Games-Howell procedure (25). We report two-sided *p*-values and consider *p* ≤ 0.05 to be significant.

## Results

### Summary of Study Population

We received claims data for 2014 from PFK for 258,399 pediatric patients. In Table 1, we provide description of genetic categories used to classify patients. Demographic features and summary statistics for the study population are described in Table 2.

**Table 1:** Description of genetic categories.

**Category**	**Description**	**Examples (diagnoses)**
1-A	Single-gene disorders or chromosomal abnormalities	Cystic fibrosis, Down's syndrome (Trisomy 21), Phenylketonuria
1-B	Birth defects/congenital anomalies; often genetic	Cleft palate, Spina bifida, Syndactyly
2	Acquired disorders; strong genetic component/predisposition	Asthma, Cancer, Type I diabetes
3^*^	Non-genetic diagnoses	Infection, Trauma, Routine health check

* *Includes all children that were not categorized into 1-A, 1-B, or 2; this is a “catch-all” category*.

**Table 2:** Demographic features and summary statistics of study population.

**Population size**	**Age: mean ±** **SD, years**		
*N* = 258,399	8.7 ± 5.1		
Features	***n*** **(%)**		
Age distribution, years			
<1	1,413 (0.55%)		
1–4	64,287 (24.9%)		
5–9	80,477 (31.1%)		
10–14	67,971 (26.3%)		
15–18	44,251 (17.1%)		
Sex: female/male	126,460 (48.9%)/131,939 (51.1%)		
Category	*n* (%)	Annual costs^*^ (Mean; 95% CI)	Annual number visits^**^ (Mean; 95% CI)
Category 1-A	11,672 (4.5%)	($4709; $4188–$5229)	(7.42; 7.30–7.53)
Category 1-B	47,090 (18.2%)	($2251; $2102–$2400)	(6.81; 6.76–6.86)
Category 2	60,307 (23.3%)	($1799; $1736–$1862)	(6.60; 6.56–6.64)
Category 3	139,330 (53.9%)	($786; $772–$800)	(4.23; 4.21–4.25)

* *costs here are the dollar amounts of paid claims, as paid by PFK*.

** *includes IP, OP-office, and ER*.

*CI, confidence interval; SD, standard deviation*.

The mean (±SD) age of patients, using oldest age in 2014, was 8.7 ± 5.1. The population was 51.1% male and 48.9% female. Overall, 58,762 (22.7%) patients were category 1 (*n* = 11,672 as 1-A and *n* = 47,090 as 1-B). Category 2 included 60,307 (23.3%) patients, while the remaining patients (*n* = 139,330; 53.9%) were category 3. The claims data encompassed 6,962 IP hospitalizations (admissions from the ER), 230,384 OP-ER visits, and 792,129 OP office visits.

Overall, the mean 2014 annual cost per 1-A child ($4,709) was nearly six times greater than category 3 children ($786). Annual costs for 1-A children were significantly higher than 1-B children, which were significantly higher than category 2, while category 3 had the lowest costs (Table 3A). We observed this significant, stepwise decrease in paid amounts and number of visits across the four categories in most of our analyses (Table 3).

**Table 3:** Summary of annual costs and healthcare utilization.

**Cost category**	**Resource utilization metric**					
		**1-A** **(*n* = 11,672)**	**1-B** **(*n* = 47,090)**	**2** **(*n* = 60,307)**	**3** **(*n* = 139,330)**	**All children** **(*N* = 258,399)**
A. All 2014 costs	Annual cost per child (USD)	$4,709	$2,251	$1,799	$786	$1,467
	Percentage of children with costs (%)	96.8%	96.3%	96.2%	87.9%	91.8%
B. Inpatient costs (including ER)	Annual cost per child (USD)	$1,594	$656	$294	$88	$307
	Percentage of children with costs (%)	6.1%	3.8%	2.8%	1.1%	2.2%
	Admissions for children with costs (# Visits)	1.49	1.26	1.20	1.08	1.22
	Cost per admission for children with costs (USD)	$17,457	$13,668	$8,746	$7,595	$11,411
	Length of stay (Days)	5.1	4.1	3.5	3.8	4.0
C. Outpatient costs (Office)	Annual cost per child (USD)	$560	$389	$329	$181	$271
	Percentage of children with costs (%)	88.9%	88.0%	85.6%	70.9%	78.3%
	Visits for children with costs (# Visits)	5.3	4.8	4.4	3.2	3.9
	Cost per visit for children with costs (USD)	$120	$93	$87	$81	$88
Outpatient costs (ER)	Annual cost per child (USD)	$238	$197	$197	$108	$151
	Percentage of children with costs (%)	54.5%	52.0%	50.5%	37.1%	43.7%
	Visits for children with costs (# Visits)	2.4	2.3	2.2	1.8	2.0
	Cost per visit for children with costs (USD)	$185	$169	$178	$161	$169
D. Prescription costs	Annual cost per child (USD)	$1,370	$437	$541	$177	$363
E. Other costs	Annual cost per subject (USD)	$947	$572	$437	$232	$374

*(All monetary values (“costs”) used for analysis are the dollar amount of paid claims, as paid by PFK. “Children with costs” is used here to describe children from each category that acquired healthcare costs in the year 2014; that is, children without costs were excluded from analyses where noted. Mean values are reported. Underlined values denote non-significance between the pair; means that do not share an underline are significantly different from each other at a level of p < 0.05. The Games & Howell procedure was used for pairwise comparisons. USD, United States dollars*.

### Inpatient Costs

IP costs were calculated by summing paid claims from facility claims and corresponding professional claims data (Table 3B). IP claims include scheduled admissions as well as ER visits resulting in hospitalization. Here, ER may also refer to urgent care claims, as they are labeled the same in PFK claims database.

**Figure 2:**
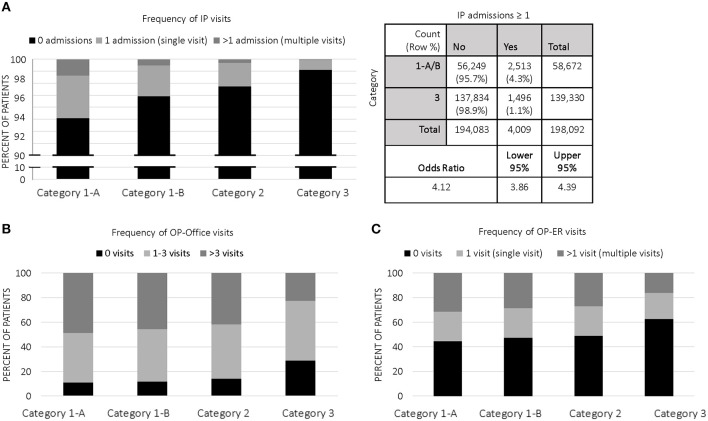
Categorical Distribution of Visits: **(A)** Frequency of IP visits among categories in 2014; 2 × 2 contingency table with odds ratio of category 1 (A&B, genetic) vs. category 3 (non-genetic); **(B)** Frequency of OP-office visits among categories in 2014; **(C)** Frequency of OP-ER visits among categories in 2014.

The proportion of children who acquired IP costs in 2014 significantly decreased from category 1 to category 3; in fact, 6.1% of 1-A children accumulated IP charges in 2014, compared to 3.8% (category 1-B), 2.8% (category 2), and 1.1% (category 3). While >97% of our study population did not experience IP admissions, the frequency of patients having at least one admission decreased in a stepwise fashion from category 1 to category 3 (Figure 2A). The odds ratio (OR) of category 1 (A&B) genetic patients experiencing ≥1 admission was OR = 4.12 (95% CI:3.86–4.39, *p* < 0.0001), compared to category 3 non-genetic patients (Figure 2A).

Specifically, children in 1-A averaged 1.5 IP visits in 2014 vs. 1.2 visits (*p* < 0.0001) for all other children in our study population. 1-A patients had average paid claims of $17,457 per admission, which was nearly 28% higher than other genetic patients in 1-B ($13,668 per admission); 1-A and 1-B were both significantly more costly for IP admissions than categories 2 ($8746) and 3 ($7595). Average LOS for IP admissions in 1-A (5.1 days; CI:4.3–5.8) was significantly higher than 1-B (4.1 days; CI:3.7–4.5), category 2 (3.5 days; CI:3.3–3.7), and category 3 (3.8 days; CI:3.2–4.4).

### Outpatient Costs

OP office costs were calculated by summing paid claims in the data flagged as “office visit.” We included scheduled office visits except for dental, vision, and mental health. Additionally, we included all costs (e.g., therapies, diagnostic testing) associated with the visit. We identified OP visits flagged as “ER” and included all associated charges with the ER visit and separate our analyses into OP office and OP-ER visits, where OP-ER visits indicate ER visits not resulting in admission (Table 3C).

### Outpatient Office

Annual mean costs for OP office visits per child were highest in 1-A ($560) and consistently decreased across all categories, with lowest costs in category 3 children ($181). The proportion of children who accumulated OP office costs in category 1 was ~88% (both 1-A and 1-B), significantly higher than the proportion of category 2 (~86%) and category 3 (~71%) who acquired OP office costs. Accordingly, the frequency of patients having at least one outpatient office visit decreased in a stepwise fashion from category 1 to category 3 (Figure 2B). Children with single gene disease and chromosomal disorders (1-A) averaged 5.3 visits annually, significantly higher than the average of our entire study population at 3.9 visits. On a per visit cost basis, OP office visit paid claims were highest in 1-A at $120, a price significantly higher than other categories.

### Outpatient ER

We observed similar trends in OP-ER data as we did in OP office data (Table 3C and Figure 2C). For example, category 1-A patients accounted for higher annual costs, higher number of annual visits, and higher cost per visit, compared to all others. Specifically, 54.5% of 1-A patients had at least one OP-ER visit, compared to 43.7% of our entire study population. The annual OP-ER paid amounts per child was highest in 1-A ($238), identical in 1-B and 2 ($197), and lowest in category 3 ($108).

### Prescription Costs

Average Rx paid amounts per child in 2014 was highest for 1-A patients at $1370, significantly higher than the average for all children in our study population ($363), and significantly more than any other category (Table 3D). Rx claims data only includes paid amounts for OP medications.

### Other Costs

In Table 3E, we display “other costs” to include paid amounts from our data not categorized as IP, OP office, OP-ER, or Rx costs. These costs may include home health visits, OP surgeries, dental, or vision care, to name a few. Although these costs come from varied categories of charge, it is of interest to note we observed significant differences between the categories of genetic patients, with the highest paid amounts in 1-A patients.

### Children Without Costs

Overall, 8.2% (*n* = 21,303) of our population did not have any claims filed for 2014; that is, their 2014 costs were zero, even though they had continuous Medicaid coverage for the entire year. We did, however, categorize these patients based on ICD-9 diagnoses from previous years and included them in some analyses as described in our study design methods section. Of the 21,303 children without any filed claims, the majority (*n* = 16,886; 79.3%) were category 3, or non-genetic patients.

### Most Common and Highest Cost ICD-9 Diagnoses

We investigated which specific ICD-9 codes per category affected the most patients (i.e., most common, Table 4) and which diagnoses were associated with the highest costs per child (Table 5). Although not the focus of this study, it would be of interest to know if there are specific genetic conditions significantly driving costs, which may help ACOs to initially target these patients for disease management and care coordination. The most common genetic diagnoses were hereditary hemolytic anemias, for example sickle cell disease (1-A) and symptoms concerning nutrition and development, for example failure to thrive (1-B), which often have significant developmental and genetic factors. The most common category 2 condition was asthma, while the most common category 3 ICD-9 code was V06 (i.e., childhood vaccinations). As expected, these most common codes were associated with highest costs in 2014 overall, given the high number of patients attributed to each code. For example, 1,227 patients in our study population received a diagnosis of cystic fibrosis (1-A) and were associated with >$19M in paid claims. When normalized to “cost per child,” the costliest genetic disorders were aplastic anemias, for example Fanconi anemia (1-A), and disorders of parathyroid gland (1-B). Among category 2, malignant neoplasms, particularly of bone and articular cartilage, were associated with highest costs on a per child basis. In fact, malignant neoplasms in general accounted for four of the top five most expensive category 2 diagnoses. Among non-genetic diagnoses in category 3, phlebitis and thrombophlebitis carried the highest costs per child.

**Table 4:** Clinical profile (ICD-9 Diagnoses) of children per category.

**Category**	**1-A**	**1-B**	**2**	**3**
Most common ICD-9 diagnoses	1. (282) Hereditary hemolytic anemias (e.g., Sickle-cell disease) 2. (271) Disorders of carbohydrate transport and metabolism 3. (758) Chromosomal anomalies (e.g., Down's syndrome) 4. (277) Unspecified disorders of metabolism (e.g., Cystic fibrosis) 5. (759) Unspecified congenital anomalies (e.g., Fragile X syndrome)	1. (783) Symptoms concerning nutrition and development (e.g., failure to thrive) 2. (754) Congenital musculoskeletal deformities (e.g., congenital pes planus) 3. (752) Congenital anomalies of genital organs (e.g., ectopic testis) 4. (757) Congenital anomalies of integument (e.g., port-wine stain) 5. (732) Osteochondropathies (e.g., juvenile osteochondrosis)	1. (493) Asthma 2. (531) Gastric ulcer 3. (216) Benign neoplasm of skin (e.g., dermatofibroma) 4. (250) Diabetes mellitus (Type I diabetes) 5. (299) Pervasive developmental disorders (e.g., autism)	1. (V06) Child vaccinations, combinations of diseases (e.g., MMR vaccine) 2. (V20) Health supervision of child 3. (V05) Child vaccinations, single disease (e.g., varicella vaccine) 4. (V04) Vaccination, specific diseases (e.g., influenza vaccine) 5. (780) General symptoms (e.g., fever)

**Table 5:** Highest annual costs^*^ ICD-9 diagnoses.

**Category**	**1-A**	**1-B**	**2**	**3**
Highest cost ICD-9 codes	1. (277) Unspecified disorders of metabolism (e.g., Cystic fibrosis) 2. (282) Hereditary hemolytic anemias (e.g., Sickle-cell disease) 3. (758) Chromosomal anomalies (e.g., Down's syndrome) 4. (759) Unspecified congenital anomalies (e.g., Fragile X syndrome) 5. (271) Disorders of carbohydrate transport and metabolism	1. (746) Other congenital anomalies of heart (e.g., congenital mitral stenosis) 2. (783) Symptoms concerning nutrition and development (e.g., failure to thrive) 3. (754) Congenital musculoskeletal deformities (e.g., congenital pes planus) 4. (745) Anomalies of cardiac septal closure (e.g., ventricular septal defect) 5. (747) Other congenital anomalies of circulatory system (e.g., patent ductus arteriosus)	1. (493) Asthma 2. (250) Diabetes mellitus (Type I diabetes) 3. (530) Diseases of esophagus (e.g., Gastroesophageal reflux disease; GERD) 4. (216) Benign neoplasm of skin (e.g., dermatofibroma) 5. (299) Pervasive developmental disorders (e.g., autism)	1. (V06) Child vaccinations, combinations of diseases (e.g., measles, mumps, and rubella vaccine; MMR vaccine) 2. (V04) Vaccination, specific diseases (e.g., influenza vaccine) 3. (V20) Health supervision of child 4. (780) General symptoms (e.g., fever) 5. (V05) Child vaccinations, single disease (e.g., varicella vaccine)

* *Analyzed cost per child for each ICD-9 dx*.

### Manual Review of Electronic Health Records

**Figure 3:**
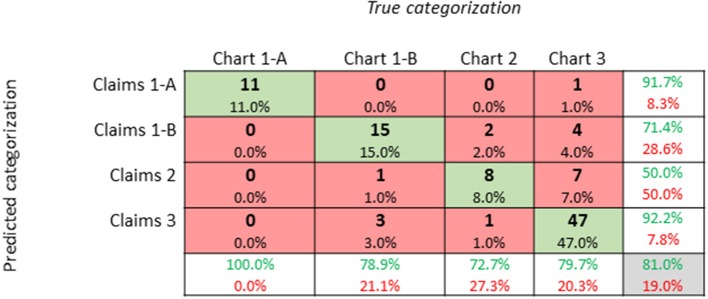
Confusion Matrix of Genetic Categorization Classification Accuracy: A confusion matrix of genetic categorization classifications from a random sampling of 100 patients. Rows correspond to the “predicted” genetic categorization for each patient based on insurance claims data. Columns correspond to the “true” categorization based on a manual review of patient electronic health charts. The diagonal cells shaded in green correspond to observations that are correctly classified, while the non-diagonal cells shaded in red correspond to incorrect observations. The column on the far right shows percentages of claims-based categorizations that are correctly (green text; positive predictive value) and incorrectly (red text; false discovery rate) classified. The row at the bottom shows percentages of chart-based categorizations that are correctly (green text; true positive rate) and incorrectly (red text; false negative rate) classified. The cell in the bottom right shows the overall accuracy (81%) of our categorization method.

For our study population, we aimed to measure the classification accuracy of our genetic categorization method (Figure 3). We sampled 100 patients to confirm if the genetic categorization based on claims data (i.e., predicted categorization) could be validated in their electronic health chart (i.e., true categorization). First, we determined four cost quantiles for all patients and then randomly selected 25 patients from each quantile. Next, for all 100 patients, we manually searched electronic health charts to categorize patients based on the categories described in Table 1. We observed agreement between claims data and electronic health records in 81/100 patients. In other words, we confirmed our claims-based genetic categorization (1-A, 1-B, 2, or 3) in 81% of our study population using health chart records.

## Discussion

The mission of an ACO is to improve the value of care, by reducing cost while either maintaining or improving the quality of clinical care. Here, we show pediatric ACOs may need to focus efforts on enrollees with genetic conditions to achieve better healthcare at lower costs. We used medical claims data to specifically report on the financial impact of genetic disease on a pediatric ACO. We describe the distribution of healthcare resource allocation of specific groups of genetic diseases, which may enable discussions of future policies, and risk adjustment based on the presence of genetic disease in children cared for within an ACO.

Our study is timely, particularly in an era when the etiologies and pathogenesis of genetic diseases are being further elucidated by the use of advancing technologies and genetic tests in a clinical setting and in large research studies. Some diseases regarded as common and complex may become reclassified as strongly genetic, as technological advances and research uncover genetic etiologies of diseases, which may include conditions such as intellectual disability, autism spectrum disorders, and other neurologic, and psychiatric disorders. We expect, therefore, many diseases will reclassify into category 1 or 2 and future studies may reveal even higher cost of healthcare and higher utilization rates attributed to genetic disease (16).

In particular, the decreasing costs of whole genome/exome sequencing (WGS/WES) has resulted in an increased use of this technology for clinical purposes. WGS/WES has led to an increased diagnostic yield in patients with suspected rare genetic conditions and in some cases, molecular diagnosis results in better prognosis and recommendations for surveillance of disease-related complications (17, 26, 27). Although the financial implications associated with WGS/WES may be costly, utilizing this technology in patients with undiagnosed or suspected genetic diseases, who may otherwise go through lengthy, costly, and unfruitful diagnostic tests, may be expected to not only improve quality of patient care but also yield cost-saving returns in the long term, given the exceptionally high cost of these patients as is (28, 29). Pediatric ACOs should be in position to drive the reimbursement policies of WGS/WES and the guidance on the best practice on the utilization of WGS/WES.

Upon designing genetic categories for our methods, we decided to label children as 1-A, 1-B, 2, and 3, rather than labeling as categories 1, 2, 3, and 4. Based on our genetics knowledge and previous studies similar to ours, we felt it sometimes necessary to make overall conclusions and statements using “category 1” as a whole including A&B, for example as we state in our abstract. We chose, however, to separate single gene and chromosomal disorders (1-A) from birth defects and congenital anomalies (1-B) given that the cause of 1-A is strictly and “wholly” genetic while 1-B may be viewed as “partially” genetic in terms of etiology.

One limitation of our study is our use of electronic database searches of computerized ICD-9 codes in insurance claims data. This type of study is highly dependent on insurance coding practices and is likely to contain some discrepancies. Therefore, we sought to determine the classification accuracy with which patient diagnoses obtained from our insurance claims data compared with manual review of electronic health chart records. To do this, we randomly selected 25 patients from each cost quantile in our dataset. Using cost quantiles allowed us to randomly select patients without any considerations of patient genetic categorization. Our manual chart review revealed that our classification method was correct in a large majority (81%) of patients. While our method of using claims data to categorize patients has an error rate, it is automated and the most appropriate method for analyzing such a large sample size in our study population (*N* = 258,399). Because most information in electronic health charts is stored in unstructured clinical notes, it is not feasible to perform such a search and categorization of all patients at once, as we were able to do with claims data.

Another limitation of the current study is that we excluded patients without a full 12 months of Medicaid enrollment eligibility in 2014, with the objective of keeping calculations and volumes of data consistent. This, however, inadvertently excluded children born after January 2014 and therefore excluded most infants from our study. Although not part of this study, it would be of interest to analyze claims data from newborns with and without genetic diseases and compare their overall costs and healthcare utilization. However, the results may vary widely because of outliers, given the often long-term and expensive neonatal intensive care unit stays for premature babies regardless of underlying genetic cause (30, 31).

## Conclusion

We found in our study population within a pediatric ACO, 22.7% of children were diagnosed with a known single gene disorder, chromosomal abnormality, or birth defect/congenital anomaly and that- collectively- these patients accounted for nearly half (42.5%) of all healthcare costs in 2014. We demonstrate patients with genetic disorders, specifically single gene and chromosomal abnormalities, had substantially higher healthcare paid claims than any other category of patients. We conclude the cost of healthcare for children with genetic diseases places a significant financial burden on PFK, indicating a need for reconsideration of the financing model of medical health care within an ACO. For example, as we continue the transition to value-based payments and risk-based contracts, pediatric ACOs could invest in increasingly cheaper genetic screening as a tool to identify and prioritize enrollees before their conditions advance to more costly states. This, in turn, implicates the importance and continued need of research of human genetic diseases. Diagnostic methods, disease interventions, and treatments that rapidly and effectively address genetic disorders should be sought after to reduce the frequency and duration of hospitalizations. Continued investment in research, applying knowledge in clinical practice, and adapting financial models will reduce not only financial impact to an ACO, but more importantly reduce the emotional and physical burdens to the patients and their families.

## Data Availability Statement

The datasets generated for this study are available on request to the corresponding author.

## Ethics Statement

The studies involving human participants were reviewed and approved by Institutional Review Board, Nationwide Children's Hospital. Written informed consent from the participants' legal guardian/next of kin was not required to participate in this study in accordance with the national legislation and the institutional requirements.

## Author Contributions

KM, YH, and SL contributed substantially to study conception and design, acquisition of data, analysis and interpretation of data, and drafting the article or revising it critically for important intellectual content. RH, SR, and RD contributed to analysis and interpretation of data. All authors have read and approved the final version of this manuscript submitted for publication.

### Conflict of Interest

The authors declare that the research was conducted in the absence of any commercial or financial relationships that could be construed as a potential conflict of interest.

## Abbreviations

ACO: accountable care organization
CI: confidence interval
ER: emergency room
ICD: international classification of diseases
IP: inpatient
IRB: institutional review board
LOS: length of stay
NCH: Nationwide Children's Hospital
OP: outpatient
PFK: Partners for Kids
Rx: Prescription
SD: standard deviation
USD: United States dollars
WGS/WES: whole genome/exome sequencing.
