# Supplementary material for: The Financial Impact of Genetic Diseases in a Pediatric Accountable Care Organization
Source: Front Public Health. 2020 Feb 28;8:58. doi: 10.3389/fpubh.2020.00058 (PMC7059305; doi:10.3389/fpubh.2020.00058)
Supplement: Supplementary file 1 [file Table_1.DOCX]

**Supplementary Table 1.** List of ICD-9 codes used for categorization

ICD-9 codes used for classifying patients into genetic categories

| ***Genetic Category*** | ***Category description*** | ***ICD-9 codes used*** | ***ICD-9 description*** |
| --- | --- | --- | --- |
| **Category 1-A** | Single-gene disorders or chromosomal abnormalities |  |  |
|  |  | 237.7X | Neurofibromatosis |
|  |  | 253.2 | Panhypopituitarism |
|  |  | 253.3 | Pituitary dwarfism |
|  |  | 255.2 | Adrenogenital disorders |
|  |  | 258.1 | Lloyd’s, Schmidt’s syndrome |
|  |  | 259.50-259.51 | Androgen insensitivity |
|  |  | 270.X, 271.X, 272.8, 272.9, 275.0, 275.1, 275.49 | Metabolic deficiencies |
|  |  | 277.XX | Cystic fibrosis and other metabolic disorders |
|  |  | 279.0X | Deficiency of humoral immunity |
|  |  | 279.1X | Deficiency of cell-mediated immunity |
|  |  | 279.2 | Combined immunity deficiency |
|  |  | 279.8 | Complement deficiency |
|  |  | 281.4 | Protein-deficiency anemia |
|  |  | 282.XX | Hereditary hemolytic anemias |
|  |  | 284.01, 284.09 | Constitutional aplastic anemia |
|  |  | 285.0 | Sideroblastic anemia |
|  |  | 286.0-286.4 | Coagulation defects |
|  |  | 288.1-288.2, 288.9 | Diseases of white blood cells |
|  |  | 289.6 | Familial polycythemia |
|  |  | 330.X | Cerebral degenerations, childhood |
|  |  | 333.4 | Huntington's chorea |
|  |  | 334.X | Spinocerebellar disease |
|  |  | 335.XX | Anterior horn cell disease |
|  |  | 345.6 | Infantile spasms |
|  |  | 356.X | Hereditary peripheral neuropathy |
|  |  | 359.0, 359.1, 359.2X, 359.3 | Muscular dystrophies and myotonic disorders |
|  |  | 362.70 | Hereditary retinal dystrophy |
|  |  | 363.50 | Hereditary choroidal dystrophy or atrophy |
|  |  | 368.51-368.54 | Color vision deficiencies |
|  |  | 371.50 | Corneal dystrophy |
|  |  | 377.16 | Hereditary optic atrophy |
|  |  | 426.7 | Anomalous atrioventricular excitation |
|  |  | 448.0 | Hereditary hemorrhagic telangiectasia |
|  |  | 520.0 | Anodontia |
|  |  | 588.1 | Nephrogenic diabetes insipidus |
|  |  | 628.1 | Adiposogenital dystrophy |
|  |  | 655.1X, 758.XX | Chromosomal anomalies |
|  |  | 759.5 | Tuberous sclerosis |
|  |  | 759.8X | Chromosomal anomalies |
|  |  | 774.0 | Perinatal jaundice from hereditary hemolytic anemias |
|  |  | 795.2 | Abnormal karyotype |
|  |  |  |  |
| **Category 1-B** | Birth defects/ congenital anomalies; often genetic |  |  |
|  |  | 228.XX | Hemangioma and lymphangioma |
|  |  | 243 | Congenital hypothyroidism |
|  |  | 246.1 | Dyshoromonogenic goiter |
|  |  | 252.1 | Hypoparathyroidism |
|  |  | 257.2, 257.8 | Testicular dysfunction |
|  |  | 272.7 | Lipidoses |
|  |  | 285.8 | Other anemias |
|  |  | 287.1 | Qualitative platelet defects |
|  |  | 289.7 | Methemoglobinemia |
|  |  | 343.X | Infantile cerebral palsy |
|  |  | 345.00-345.01, 345.10-345.11 | Epilepsy |
|  |  | 358.8 | Myoneural disorder |
|  |  | 359.71, 359.79, 359.89, 359.9 | Muscular dystrophies |
|  |  | 368.61 | Congenital night blindness |
|  |  | 379.51 | Congenital nsytagmus |
|  |  | 389.0 | Conductive hearing loss |
|  |  | 520.5 | Hereditary disturbances in tooth structure |
|  |  | 524.0X | Major anomalies of jaw size |
|  |  | 550.XX | Inguinal hernia |
|  |  | 553.1, 553.3 | Abdominal hernia |
|  |  | 593.7X | Vesicoureteral reflux |
|  |  | 619.X | Fistula, female genital tract |
|  |  | 654.0X | Congenital abnormalities of uterus |
|  |  | 703.8 | Other specified diseases of nail |
|  |  | 728.3, 728.5 | Disorders of muscle, ligment, fascia |
|  |  | 732.X | Osteochondropathies |
|  |  | 740.X | Anencephalus |
|  |  | 741.XX | Spina bifida |
|  |  | 742.XX, 743.XX, 744.XX, 745.XX, 746.XX, 747.XX, 748.XX, 750.XX, 751.XX, 752.XX, 753.XX, 754.XX, 755.XX, 756.XX, 757.XX, 759.X, 778.6 | Congenital anomalies |
|  |  | 749.XX | Cleft palate and cleft lip |
|  |  | 783.41 | Failure to thrive |
|  |  |  |  |
| **Category 2** | Acquired disorders; strong genetic component/ predisposition |  |  |
|  |  | 140.XX-239.XX | Neoplasms |
|  |  | 242.00-242.01 | Graves’ disease |
|  |  | 244.9 | Primary hypothyroidism |
|  |  | 250.XX | Diabetes mellitus |
|  |  | 299.00-299.01 | Developmental disorders |
|  |  | 307.23 | Tourette's disorder |
|  |  | 346.0X, 346.3X | Migraine |
|  |  | 376.42 | Exostosis of orbit |
|  |  | 380.81 | Exostosis of ear canal |
|  |  | 437.5 | Moyamoya disease |
|  |  | 471.X | Nasal polyps |
|  |  | 493.XX | Asthma |
|  |  | 530.81 | Gastroesophageal reflux disease |
|  |  | 536.2 | Persistent vomiting |
|  |  | 555.0-555.9 | Crohn's disease |
|  |  | 556.X | Ulcerative colitis |
|  |  | 579.0 | Celiac disease |
|  |  | 581.XX | Nephrotic syndrome |
|  |  | 582.XX | Chronic glomerulonephritis |
|  |  | 583.XX | Nephritis and nephropathy |
|  |  | 585.6 | End stage renal disease |
|  |  | 591 | Hydronephrosis |
|  |  | 617.X | Endometriosis |
|  |  | 620.0-620.2 | Ovarian cysts |
|  |  | 704.0X | Alopecia |
|  |  | 710.0 | Systemic lupus erythematosus |
|  |  | 710.1 | Systemic sclerosis |
|  |  | 710.3 | Dermatomyositis |
|  |  | 710.4 | Polymyositis |
|  |  | 714.0 | Rheumatoid arthritis |
|  |  | 714.3X | Juvenile chronic polyarthritis |
|  |  | 718.3X | Recurrent dislocation of joint |
|  |  | 733.2X | Cyst of bone |
|  |  | 737.0 | Adolescent postural kyphosis |
|  |  | 791.3 | Myoglobinuria |
|  |  |  |  |
